# Cerebellar white and gray matter abnormalities in temporal lobe epilepsy: a voxel-based morphometry study

**DOI:** 10.3389/fnins.2024.1417342

**Published:** 2024-08-01

**Authors:** Yini Chen, Jingyu Pan, Andong Lin, Lu Sun, Yufei Li, Hongsen Lin, Renwang Pu, Ying Wang, Yiwei Qi, Bo Sun

**Affiliations:** ^1^Department of Radiology, The First Affiliated Hospital of Dalian Medical University, Dalian, China; ^2^Department of Neurology, The First Affiliated Hospital, Dalian Medical University, Dalian, China; ^3^Department of Neurology, Taizhou Municipal Hospital, Taizhou, China

**Keywords:** temporal lobe epilepsy, VBM, cerebellum, cognitive, MMSE

## Abstract

**Background:**

Previous structural neuroimaging studies linked cerebellar deficits to temporal lobe epilepsy (TLE). The functions of various cerebellar regions are increasingly being valued, and their changes in TLE patients warrant further in-depth investigation. In this study, we used the Spatially Unbiased Infratentorial (SUIT) toolbox with a new template to evaluate the cerebellar structural abnormalities in patients with TLE, and further explored the relationship between the changes of different cerebellar regions and cognition.

**Methods:**

Thirty-two patients with TLE were compared with 39 healthy controls (HC) matched according to age, gender, handedness, and education level. All participants underwent a high-resolution T1-weighted MRI scan on a 3.0 Tesla scanner. We used a voxel-based morphometry (VBM) approach utilizing the SUIT toolbox to provide an optimized and fine-grained exploration of cerebellar structural alterations associated with TLE.

**Results:**

Compared with HC, TLE patients showed a significant reduction in the volume of gray matter in the Left lobule VI and white matter in the Right Crus II. In the TLE patient group, we conducted partial correlation analysis between the volumes of different cerebellar regions and cognitive rating scale scores, such as MMSE and MoCA. The volume of the Left lobule VI (GM) exhibited a positive correlation with the MMSE score, but no significant correlation was found with the MoCA score. Furthermore, it was observed that the MMSE was more effective than the MoCA in identifying epilepsy patients with cognitive impairment.

**Conclusion:**

This study supported previous research indicating that temporal lobe epilepsy (TLE) is linked to structural changes in the cerebellum, specifically affecting the volume of gray matter. These findings offer valuable insights into the neurobiology of TLE and hold potential to inform the development of enhanced diagnostic methods and more effective treatment approaches.

## Introduction

Epilepsy, characterized by repeated and unanticipated seizures, is a common chronic neurological disease (Rossini et al., 2021). Temporal Lobe Epilepsy (TLE), the most prevalent subtype of focal epilepsy in adults, is frequently associated with alterations in neuronal structures, including both cell body and synaptic injury (Singh and Trevick, 2016; Rossini et al., 2021). Previous studies at the electrophysiological, molecular, and behavioral levels have extensively described the pathological mechanisms of TLE (Tallarita et al., 2019; Elsherif and Esmael, 2021; Peplow and Martinez, 2023). However, the role gray and white matter abnormalities play in TLE, particularly how structural changes impact cognitive functionality in patients, warrants further research.

The cerebellum, an integral structure of the human brain, was implicated in the regulation of various functions, including cognition, movement, memory, emotion, and mood (Sokolov et al., 2017; Ashida et al., 2018; Jacobi et al., 2021; Ferrari et al., 2022; Petrosini et al., 2022). Recently, increasing numbers of neuroscientific investigations had begun to focus on the cerebellum in their study of neural disorders (Jacobs et al., 2018; Huang et al., 2021; Li et al., 2023; Rubio et al., 2023; Iskusnykh et al., 2024). TLE being the most common subtype of epilepsy, numerous researches had been conducted on its mechanism of onset, clinical manifestations, imaging characteristics, structural differences, and functional abnormalities (Yang et al., 2012; Osipowicz et al., 2016; Yang et al., 2020; Sandhu et al., 2021; Park et al., 2022). Voxel-Based Morphometry (VBM), a technique for comprehensive, automated, and objective analysis of brain structure based on voxel magnetic resonance imaging, enabled precise morphological study of the living brain. VBM quantitatively calculated and analyzed the changes in the density or volume of cerebellum and white matter in each voxel of MRI to reflect the corresponding anatomical structure differences, making it one of the most common methods to evaluate cerebral gray matter and white matter lesions (Höflich et al., 2017). TLE, a neurodevelopmental disorder, typically involves subcortical volume loss, cortical atrophy, and white matter disruption. Numerous studies based on VBM analysis of TLE patient structural images had proved that the longer the disease course and higher the seizure frequency, the more severe the gray matter atrophy (Bonilha et al., 2006; Coan et al., 2009). In addition to gray matter, white matter also underwent varying degrees of atrophy due to epileptic seizures (Keller, 2002; Ballerini et al., 2023).

The cerebellum’s structural (and functional) changes appeared to be consistent across different species and studies, yet they have not garnered much attention in human studies. This may be due to methodological issues to some extent. Most structural image analysis studies based on 3D T1-weighted Images (3D T1WI) solely focus on supratentorial brain structures, and tools widely used for assessing cortical thickness, such as Freesurfer, do not include the cerebellum. For VBM method, the small size of functional areas in the cerebellum posed unique challenges to inter-subject standardization, and its results were dependent on the accurate standardization of the cerebellum (Diedrichsen et al., 2010). Therefore, conventional whole-brain-based VBM had proven to be suboptimal for cerebellar analysis (Diedrichsen, 2006; Diedrichsen et al., 2009). To address these issues, our study introduces the Spatially Unbiased Infratentorial (SUIT) for more accurate analysis of cerebellar structures. SUIT maintained lesser spatial differences between individuals and retained anatomical features, providing a more accurate alignment between subjects compared to whole-brain methods by conducting a detailed analysis of cerebellar subregions (three lobes and lobules I-X) using automatic nonlinear normalization. Furthermore, the compatibility of SUIT with popular brain imaging analysis tools like Statistical Parametric Mapping (SPM) taken into consideration during its design, facilitating researchers to perform data analysis using existing tool chains. Previous studies had effectively utilized SUIT to identify morphological changes in cerebellar subregions (Kühn et al., 2011; Piccinin et al., 2014; Hirjak et al., 2015). In comparison to segmentation techniques like FSL, SPM, FreeSurfer, and ACAPULCO, the specialized templates offered by SUIT exhibit markedly superior performance in examining deep nuclei. This enhanced capability facilitates a more detailed investigation of potential pathological alterations in these specific nuclei (Bonilha et al., 2006; Harding et al., 2022). This prominent advantage significantly contributes to an in-depth investigation of cerebellar changes in temporal lobe epilepsy.

The role of the cerebellum in epilepsy had gained an increasing amount of attention due to detailed elaboration on neuro-pathological variations associated with it (Rondi-Reig, 2022; Rubio et al., 2023). TLE, the most common type of epilepsy, had been widely researched (Keller, 2002; Mueller et al., 2006; Park et al., 2022; Peplow and Martinez, 2023). Increasing lines of evidence have implicated that the cerebellum played a role in cognitive processing as well as sensorimotor control through the cerebro-cerebellar circuit (Guo et al., 2021; Wang et al., 2023). Structural image analyses on TLE patients have primarily focused on the cerebral changes, with a conspicuous absence in the exploration of structural alternations in the subtentorial cerebellum. This untouched area of research shows promise, as therapies promoting cerebellar stimulation have demonstrated significant therapeutic benefits in treating seizures (Ming et al., 2021). This revelation could present a new pathway for treatment. A recent large-scale, multicenter study examining epilepsy and cerebellar involvement has identified patterns of cerebellar atrophy across various epilepsy types. These findings indicate more extensive pathological alterations in the cerebellum. However, the study did not delve into changes within the three pairs of deep nuclei (Kerestes et al., 2023). Therefore, this study adopts the SUIT toolbox for a refined investigation into the structural changes in the cerebellum of TLE patients. Furthermore, the research aims to discern any correlation between these abnormal cerebral volumes and the cognitive scores recorded in TLE patients.

## Patients and methods

### Participants

All patients were consistently diagnosed by a senior neurologist with 16 years of experience, adhering to the 2017 diagnosis criteria for focal epilepsy set by the International League Against Epilepsy (ILAE) (Fisher et al., 2005). The diagnosis integrated information from medical history, physical examination, electroencephalogram results, Magnetic Resonance Imaging (MRI) results, and laboratory tests. An experienced neuro-diagnostics physician with 17 years of service independently reviewed each patient’s MRI images. This review ensured the exclusion of conditions such as deformities, tumors, reactive gliosis, and other epilepsy-causing alterations. Any past brain surgeries, chronic internal diseases unrelated to epilepsy, MRI contraindications, history of drug abuse, or psychiatric history were also excluded from the study. In summary, this study retrospectively collected a total of 66 TLE patients who met the ILAE criteria from January 2021 to January 2024, including 6 cases of encephalitis and 28 cases of lacunar infarction. Therefore, a final group of 32 TLE patients was included.

All participants underwent neuropsychological scales testing administered by a professional psychoanalyst. The Minimum Mental State Examination (MMSE) and the Montreal Cognitive Assessment (MoCA) were used to evaluate cognitive abilities. Meanwhile, the Hamilton Anxiety Scale (HAMA) and Hamilton Depression Scale (HAMD) were employed for psychological assessment. A total of 32 patients with temporal lobe epilepsy were included in the study. We also recruited 39 age, gender, handedness, and education level matched healthy volunteers as controls. The entire study plan was approved by the Ethics Committee of the First Affiliated Hospital of Dalian Medical University.

### Imaging data acquisition

In this study, all images were retrospectively collected from uMR Omega 3.0 T MR (United Image Healthcare, Shanghai, China) and Philips Ingenia CX 3.0 T (Philips Healthcare, Best, Netherlands) scanners. These devices, fitted with 32-channel head coils, enabled us to acquire brain MRI images. All of our subjects were scanned in a supine position, and they were instructed to maintain stillness throughout the scanning procedure. The 3D T1WI sequence parameters were as follows: For the uMR Omega, repetition time = 9.0 ms, echo time = 3.6 ms, voxel size = 0.5 × 0.5 × 0.5 mm^3^, slice thickness = 0.5 mm, matrix size = 512 × 360, and number of slices = 440. For the Philips Ingenia, repetition time = 8.4 ms, echo time = 3.8 ms, voxel size = 1 × 1 × 1 mm^3^, matrix size = 200 × 200, and number of slices = 220. The structural images derived from these scans served the essential purpose of identifying and ruling out possible intracranial structural abnormalities such as severe white matter lesions, cerebrovascular conditions, brain atrophy, cerebral infarction, ectopic gray matter, and cerebral hemorrhages.

### Imaging processing

First, two senior imaging diagnosticians examined the T1WI images of each subject and marked those with poor image quality (false artifacts and incomplete images) and parenchymal lesions for exclusion. After checking for scanner artifacts and gross anatomical abnormalities, and setting the image origin at the anterior commissure in each subject, we isolated the infratentorial structures (i.e., cerebellum and brainstem) from the surrounding tissue by using the Isolate function within the SUIT toolbox (Diedrichsen et al., 2009).^1^ The isolation procedure includes the segmentation of the brain into tissue types using the unified segmentation (Ashburner and Friston, 2005) approach as implemented in MATLAB 2016b and SPM12.^2^ Where necessary, the isolated maps were hand corrected using MRIcron,^3^ excluding any tissue included outside the cerebellum or brainstem. Subsequently, each individual’s cerebellar gray and white matter segments were normalized onto the SUIT atlas template, allowing for an improved alignment of individual fissures and cerebellar subregions when compared to conventional whole-brain VBM (Diedrichsen, 2006; Diedrichsen et al., 2009). A modulation of the segmented gray and white matter probability maps was applied in order to compensate for volume changes during spatial normalization by multiplying each voxel’s intensity value with the Jacobian determinants. Before statistical analysis, all probability images were smoothed with a 4 mm full-width at half-maximum (FWHM) smoothing kernel in SPM12. This relatively small smoothing kernel was chosen to preserve precision in the definition of cerebellar substructures. The use of a 4 mm kernel is in line with previous studies that focused on the cerebellum by means of the SUIT toolbox (D’Agata et al., 2011; Kühn et al., 2012). Then, we conducted a voxelwise two-sample *T* test with sex, age, total intracranial volume (TIV) and education as covariates to compare GMV and WMV between the two groups. The threshold was *p* < 0.001, two-tailed at the voxel level, and false discovery rate (FDR)-corrected to *p* < 0.05 at the cluster level. We used Duvernoy’s Atlas of the Human Brainstem and Cerebellum to identify the locations of significantly different clusters overlaid onto the SUIT brainstem template (Diedrichsen et al., 2009).

### Partial correlation analysis

After analyzing the statistically significant differences in the cerebellum areas using SPM, the volumes of gray and white matter in these brain areas were individually extracted for both groups of subjects using DPABI_V8.1^4^ (Yan et al., 2016). Subsequently, the scale scores (MMSE and MoCA) of the TLE group patients were compiled and a partial correlation analysis was conducted, taking into account factors such as age, gender, duration of the disease, number of years of education, and TIV which were integrated as covariates in the calculation.

### Statistical analysis

In this study, we utilized R language,^5^ a free software environment for statistical computing and graphics, to analyze the demographic and clinical data across the two groups. Depending on the nature of the data, we applied either the Independent sample *t*-test or nonparametric test for intergroup comparison of measurement data. Data conforming to a normal distribution were expressed as mean ± standard deviation, whereas, for data not conforming to a normal distribution, we used the median and interquartile range to characterize them. In order to eliminate the potential influence of factors such as age, gender, years of education, and TIV, these factors were included in the analysis as covariates in this process. SPM maps were initially thresholded at *p* < 0.001, uncorrected. In order to minimize the possibility of false-positive results, only those differences or associations surviving a cluster level FDR correction (*p* < 0.05, cluster (k) threshold = 10 voxels) were considered for reporting. When conducting partial correlation analysis, age, gender, disease duration, years of education, and TIV were included as covariates in the analysis to evaluate the relationship between MoCA and MMSE scale scores and the gray and white matter volumes of different cerebellar regions in the TLE group.

## Results

### Demographic data and clinical data

Table 1 summarized the clinical data of TLE patients and the HC group. There were no significant differences in gender, age, and education level between TLE patients and the HC group (*p* > 0.05). However, significant differences were observed in MMSE, MoCA, HAMA, and HAMD scores between the two groups, with TLE patients scoring lower than the HC group in all these measures (*p* < 0.001).

**Table 1:** Clinical data of the TLE and HC groups.

Characteristics	HC (*n* = 39)	TLE (*n* = 32)	Statistic	*p*
Age at examination/year	58.00 (31.50, 64.50)	46.50 (34.75, 51.75)	Z = −1.74	0.082
Gender, *n* (%)			χ^2^ = 1.43	0.231
Male	14 (35.90)	16 (50.00)		
Female	25 (64.10)	16 (50.00)		
MMSE	28.00 (28.00, 29.00)	27.00 (24.00, 29.00)	Z = −3.33	<0.001
Moca	27.00 (25.00, 27.00)	22.00 (18.00, 26.00)	Z = −4.31	<0.001
HAMA	5.00 (4.00, 6.00)	8.00 (6.00, 12.00)	Z = −3.43	<0.001
HAMD	3.00 (2.00, 6.00)	7.50 (5.00, 10.25)	Z = −3.94	<0.001
Education/year	12.00 (9.00, 16.00)	12.00 (9.00, 15.00)	Z = −0.99	0.322
Disease duration/month	–	30.00 (10.50, 150.00)	–	–

Z, Mann–Whitney test; χ^2^, Chi-square test.

### Regional volumetric differences between TLE and HC

**Figure 1:**
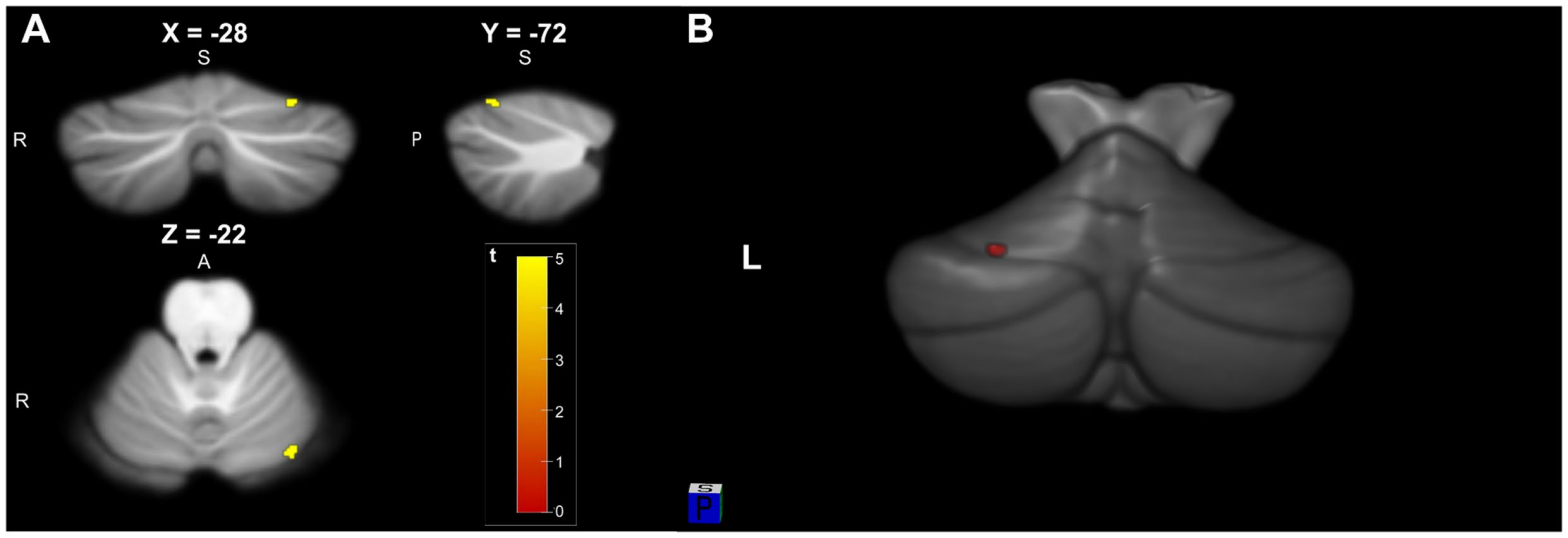
**(A)** Significant cluster of cerebellar gray matter volume reduction in Left lobule VI (−28, −72, −22) in TLE patients compared to HC (pFDR <0.05 and KE = 10). **(B)** 3D visualization model of cerebellar gray matter differential brain area superimposed on SUIT T1-weighted cerebellar template.

When compared with HC group, TLE group were characterized by a reduced cerebellar gray matter (GM) volume within the left lobule VI (*p* < 0.05, FDR) (Figure 1). After FDR correction, there were no significant differences in white matter volume between the two groups. Anatomical and statistical details of these differences are summarized in Table 2.

**Figure 2:**
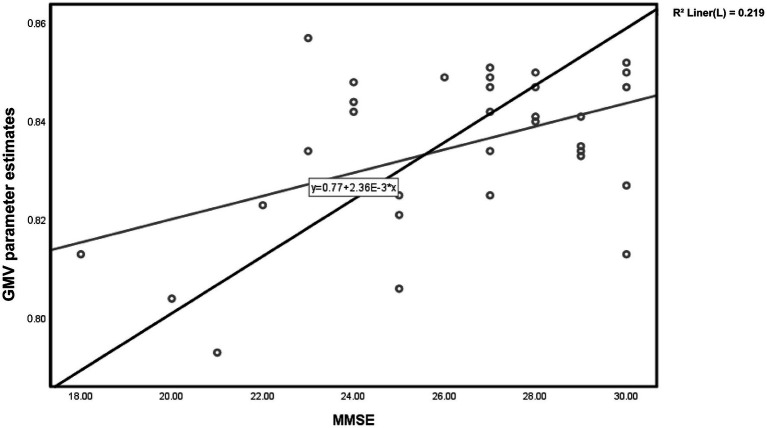
Scatter plot of voxel-wise regression analysis of MMSE scores and local gray matter volume (GMV) parameter estimates in TLE.

**Table 2:** Cerebellar measures in patients and healthy controls.

Cerebellar tissue	Comparison	Anatomical region	Cluster-level			Voxel-level					x, y, z (mm)		
			P_FWE−corr_	k_E_	P_uncorrected_	P_FWE−corr_	P_FDR−corr_	T	(Z_三_)	P_uncorrected_			
GM	HC > TLE	Left lobule VI	0.012	40	0.054	0.021	0.039	5.28	4.80	0.000	−28	−72	−22

### Correlation of scale score with regional cerebellar morphology

For patients in the TLE group, partial correlation analysis demonstrated a significant positive correlation between the gray matter volume in the Left lobule VI and MMSE scores (*r* = 0.517, *p* = 0.006) (Figure 2). However, there was no significant correlation observed with the MoCA scores (*r* = 0.325, *p* = 0.098).

### Disease duration with regional cerebellar morphology

For patients in the TLE group, partial correlation analysis results showed that there was no significant correlation between the two aforementioned cerebellar regions and the duration of the disease. The relationship between the gray matter volume of the left cerebellar lobule VI and the duration of the disease is (*r* = 0.118, *p* = 0.550).

## Discussion

In the present MRI study, we aimed at investigating structural cerebellar differences between patients with TLE and healthy controls, and at characterizing the relationship between the MMSE scores and regional cerebellar morphology in the TLE groups. For the analysis of imaging data, a sophisticated voxel-based approach designated to allow for a detailed characterization of anatomical abnormalities in the cerebellum, was applied. Two main findings emerged: (i) TLE patients have significantly reduced cerebellar volumes with atrophy pronounced in the Left lobule VI (GM) and (ii) increased MMSE scores are related to increased volumes in Left lobule VI (GM).

In structural neuroimaging studies, cerebellar gray matter reduction in patients with TLE has been mentioned, but different cerebellar regions are not exactly the same (Oyegbile et al., 2011; Marcián et al., 2018; Wang et al., 2023). Discrepant results might be explained by aspects of sample selection and analysis methods used to characterize cerebellar morphology. In this study, the application of the SUIT toolbox enabled us to automatically isolate the cerebellum and the brainstem from the cerebral cortex in order to identify the regional localization of tissue alterations underlying TLE more precisely.

In this study, the differential cerebellar regions identified are the Left lobule VI (GM), diverging from findings of prior reports. Such disparities could stem from participant quantity, disease duration, medication usage, and various other elements. However, the significance of our work lies in underscoring the presence of differential cerebellar regions, necessitating further corroboration via increased sample sizes in succeeding investigations. Despite the relatively compact voxel size of these differential regions, it could be attributed to rigorous cybernetic parameters and multiple covariate control.

The association between reduced cerebellar volume and cognitive impairment has been firmly established (Hellwig et al., 2013). In this study, we identified a decrease in gray matter volume in the Left lobule VI of patients with TLE. We discovered a significant positive correlation between MMSE scores and the reduced volume within the Left lobule VI. The importance of this correlation can be understood in two aspects. Firstly, from an anatomical perspective, lobule VI serves as one of the anatomical foundations of cerebellar cognitive function (Buckner, 2013). Previous fMRI studies have shown that parts of the Left lobule VI are involved in language, spatial, and executive functions (Stoodley and Schmahmann, 2009; Stoodley et al., 2010). Secondly, lobule VI, located in the anterior cerebellum, has been proven to play a role in fine motor and visuomotor adaptation skills (Stoodley and Schmahmann, 2009; Stoodley et al., 2010; Bernard and Seidler, 2013a,b).

Although many current studies primarily focus on gray matter when examining structural brain abnormalities based on VBM, the differences in white matter also receive attention (Mueller et al., 2006; Oyegbile et al., 2011). Previous findings regarding cerebellar white matter changes in patients with TLE have been inconsistent. Some studies suggest that TLE patients demonstrate a reduction in cerebellar white matter volume, while others show no noticeable atrophy (Mueller et al., 2006; Oyegbile et al., 2011). In this study, after FDR correction, we did not identify any significant differences in the cerebellar white matter between the two groups. It may be necessary to continue expanding the sample size for further research.

The results of this study suggest a positive correlation between the volume of Left lobule VI and MMSE scores, but no significant association with MoCA scores. MMSE and MoCA are both commonly used scales to evaluate cognitive functions, but they have differences in their assessments. MoCA is a more detailed assessment tool than MMSE, as it evaluates not only most cognitive areas covered by MMSE, but also includes executive functions such as planning and multitasking. Therefore, MoCA performs better in detecting subtle cognitive impairments (Pendlebury et al., 2012; Biundo et al., 2016; Wu et al., 2021). However, our study found that the volume of Left lobule VI is only positively correlated with MMSE scores and not significantly associated with MoCA scores, which is surprising considering MoCA’s ability to detect subtle cognitive deficits. One possible reason for this discrepancy could be that MMSE focuses more on language and memory components, while MoCA places more emphasis on assessing executive abilities (Lou et al., 2007; Chou et al., 2014; Wu et al., 2021). Previous research has shown that patients with TLE are more likely to have language and declarative memory impairments (Sidhu et al., 2015; Bremm et al., 2019; Banjac et al., 2021), while executive abilities impairments are less common, which supports this hypothesis. Additionally, the type and duration of epilepsy in TLE patients can have an impact on these cognitive assessment scores. However, these factors often vary among individuals, which may offer another explanation for this intriguing phenomenon. Although the correlation difference between MMSE and MOCA might be statistically insignificant, this finding provides a new perspective for future research: exploring the ability of different cognitive tests to reveal specific neuropathological changes. Of course, future research must further expand the sample size to explore more deeply.

Despite controlling for covariates, our partial correlation analysis found no significant association between disease duration and cerebellar regions. Contrarily, earlier studies have reported a progressive reduction in cerebella volume with extended epilepsy duration (Bonilha et al., 2006; Kerestes et al., 2023). This apparent divergence may be attributed to the relatively small sample size used in our study, underscoring the necessity for future investigations using expanded sample sizes to definitively clarify these observations. Regrettably, one of the primary reasons for conducting cerebellar-level research using SUIT was to further investigate alterations in deep nuclei. However, the samples included in this study did not exhibit significant differences. Nonetheless, we remain confident that SUIT can aid in exploring potential changes in deep nuclei.

The complexity of medication status, which involves the use of various drugs and dosages, in the patients participating in this study ought to be acknowledged. Such factors can indeed influence changes in cerebellar volume (De Marco et al., 2003; Kerestes et al., 2023). Given this complexity, our present research did not delve profoundly into the relationship between medication use and alterations in the cerebellum.

## Limitation

First and foremost, one limitation of this study is the relatively small sample size of patients. However, to ensure the accuracy of the study, images of structural abnormalities, such as severe white matter lesions, cerebrovascular diseases, brain atrophy, brain trauma, cerebral infarction, gray matter heterotopia, and hemorrhage, must be excluded. Consequently, the number of patients eligible for inclusion in the study is restricted due to this screening process. Secondly, it is important to acknowledge that the medication administered to TLE patients during their illness may have an impact on brain functions and structures. Therefore, it is crucial to thoroughly investigate the potential effects of long-term medication on the cerebellum in TLE and take them into account in future studies. Thirdly, this study solely examines the correlation between the overall score of the cognitive assessment scale and the volume of gray and white matter in the cerebellum. Future research should concentrate on analyzing the subscales, specifically the five cognitive domains. Lastly, it is crucial to validate these findings with a larger sample size and data obtained from multiple imaging centers, as individual differences in brain structure and variations in scanners may influence the results.

## Conclusion

To the best of our knowledge, the present study is the first using a cerebellum-optimized VBM (SUIT) procedure that investigated cerebellar correlates of MMSE in TLE. Our data suggest that future research on morphological correlates of TLE might benefit from considering the cognitive cerebellum. In this respect, we strongly advocate neuroimaging studies combining both structural and functional MRI methods to further explore the subtle pathological changes of cerebellum in patients with TLE.

## Data availability statement

The original contributions presented in the study are included in the article/supplementary material, further inquiries can be directed to the corresponding authors.

## Ethics statement

The studies involving humans were approved by the First Affiliated Hospital of Dalian Medical University. The studies were conducted in accordance with the local legislation and institutional requirements. The participants provided their written informed consent to participate in this study. Written informed consent was obtained from the individual(s) for the publication of any potentially identifiable images or data included in this article.

## Author contributions

YC: Conceptualization, Data curation, Formal analysis, Investigation, Methodology, Project administration, Resources, Software, Supervision, Validation, Visualization, Writing – original draft, Writing – review & editing. JP: Conceptualization, Data curation, Formal analysis, Investigation, Methodology, Project administration, Resources, Visualization, Writing – original draft, Writing – review & editing. AL: Conceptualization, Data curation, Formal analysis, Investigation, Methodology, Software, Supervision, Validation, Visualization, Writing – original draft, Writing – review & editing. LS: Conceptualization, Data curation, Investigation, Methodology, Software, Visualization, Writing – original draft, Writing – review & editing. YL: Data curation, Methodology, Software, Supervision, Validation, Writing – original draft, Writing – review & editing. HL: Conceptualization, Data curation, Investigation, Methodology, Software, Supervision, Validation, Visualization, Writing – original draft, Writing – review & editing. RP: Supervision, Writing – original draft, Writing – review & editing, Conceptualization, Investigation, Methodology, Resources, Software. YW: Methodology, Resources, Supervision, Visualization, Writing – original draft, Writing – review & editing. YQ: Conceptualization, Data curation, Formal analysis, Investigation, Methodology, Software, Supervision, Validation, Visualization, Writing – original draft. BS: Conceptualization, Funding acquisition, Project administration, Resources, Software, Supervision, Validation, Visualization, Writing – original draft, Writing – review & editing.

## Footnotes

^1^ http://www.icn.ucl.ac.uk/motorcontrol/imaging/suit.htm

^2^ http://www.fil.ion.ucl.ac.uk/spm/

^3^ http://www.mccauslandcenter.sc.edu/mricro/mricron/index.html

^4^ https://rfmri.org/DPABI

^5^ https://www.r-project.org/
