# Corrigendum: Cerebellar white and gray matter abnormalities in temporal lobe epilepsy: a voxel-based morphometry study

**DOI:** 10.3389/fnins.2024.1546300

**Published:** 2025-01-09

**Authors:** Yini Chen, Jingyu Pan, Andong Lin, Lu Sun, Yufei Li, Hongsen Lin, Renwang Pu, Ying Wang, Yiwei Qi, Bo Sun

**Affiliations:** ^1^Department of Radiology, The First Affiliated Hospital of Dalian Medical University, Dalian, China; ^2^Department of Neurology, The First Affiliated Hospital, Dalian Medical University, Dalian, China; ^3^Department of Neurology, Taizhou Municipal Hospital, Taizhou, China

**Keywords:** temporal lobe epilepsy, VBM, cerebellum, cognitive, MMSE

In the published article, there was an error to Figure 2 as published. The original Figure 3 should be Figure 2 and the current Figure 2 should be deleted.

In the published article, there was an error in Table 2 as published. The corrected Table 2 and its caption appear below.

**Table 2:** Cerebellar measures in patients and healthy controls.

**Cerebellar tissue**	**Comparison**	**Anatomical region**	**Cluster-level**			**Voxel-level**					**x,y,z (mm)**		
			**P**_FWE − corr_	**k**_E_	**P**_uncorrected_	**P**_FWE − corr_	**P**_FDR − corr_	**T**	**(Z**_Ξ_ **)**	**P**_uncorrected_			
GM	HC > TLE	Left lobule VI	0.012	40	0.054	0.021	0.039	5.28	4.80	0.000	−28	−72	−22

In the published article, there was an error to the section **Abstract**, *Results*. This sentence previously stated:

“On the other hand, there was no significant correlation observed between the volume of the Right Crus II (WM) and the two cognitive scale scores mentioned above.”

This sentence should be deleted. The correct section **Abstract**, *Results* appears below:

“**Results:** Compared with HC, TLE patients showed a significant reduction in the volume of gray matter in the Left lobule VI and white matter in the Right Crus II. In the TLE patient group, we conducted partial correlation analysis between the volumes of different cerebellar regions and cognitive rating scale scores, such as MMSE and MoCA. The volume of the Left lobule VI (GM) exhibited a positive correlation with the MMSE score, but no significant correlation was found with the MoCA score. Furthermore, it was observed that the MMSE was more effective than the MoCA in identifying epilepsy patients with cognitive impairment.”

A correction has been made to **Abstract**, *Conclusion*. This sentence previously stated:

“This study supported previous research indicating that temporal lobe epilepsy (TLE) is linked to structural changes in the cerebellum, specifically affecting the volume of both gray and white matter.”

The corrected sentence appears below:

“This study supported previous research indicating that temporal lobe epilepsy (TLE) is linked to structural changes in the cerebellum, specifically affecting the volume of gray matter.”

In the published article, there was an error. A correction has been made to **Results**, *Regional volumetric differences between TLE and HC*. This sentence previously stated:

“Further, they showed a reduced white matter (WM) volume within the Right Crus II (*p* < 0.05, FDR) (Figure 2).”

The corrected sentence appears below:

“After FDR correction, there were no significant differences in white matter volume between the two groups.”

In the published article, there was an error. A correction has been made to **Results**, *Correlation of scale score with regional cerebellar morphology*. This sentence previously stated:

“The white matter volume in Right Crus II showed no significant association with


$$\begin{array}{r} \text{MMSE scores }\left(\text{r }=\;-0.034,\text{ p }=\;0.856\right),\\ \text{as well as with MoCA scores }\left(\text{r }=\;0.086,\text{p }=\;0.640\right).” \end{array}$$

This sentence should be deleted. The corrected section **Results**, *Correlation of scale score with regional cerebellar morphology* appears below:

“For patients in the TLE group, partial correlation analysis demonstrated a significant positive correlation between the gray matter volume in the Left lobule VI and MMSE scores (*r* = 0.517, *p* = 0.006) (Figure 2). However, there was no significant correlation observed with the MoCA scores (*r* = 0.325, *p* = 0.098).”

In the published article, there was an error. A correction has been made to **Results**, *Disease duration with regional cerebellar morphology*. This sentence previously stated:

“The relationship between the gray matter volume of the left cerebellar lobule VI and the duration of the disease is (r=0.118, p=0.550), while the relationship between the white matter volume of the right cerebellar Crus II and the duration of the disease is (r=-0.013, p=0.947).”

The corrected sentence appears below:

“The relationship between the gray matter volume of the left cerebellar lobule VI and the duration of the disease is (*r* = 0.118, *p* = 0.550).”

In the published article, there was an error. A correction has been made to **Discussion**, *First paragraph*. This sentence previously stated:

“Two main findings emerged: (i) TLE patients have significantly reduced cerebellar volumes with atrophy pronounced in the Left lobule VI (GM) and the Right Crus II (WM) and (ii) increased MMSE scores are related to increased volumes in Left lobule VI (GM).”

The corrected sentence appears below:

“Two main findings emerged: (i) TLE patients have significantly reduced cerebellar volumes with atrophy pronounced in the Left lobule VI (GM) and (ii) increased MMSE scores are related to increased volumes in Left lobule VI (GM).”

In the published article, there was an error. A correction has been made to **Discussion**, *Third paragraph*. This sentence previously stated:

“In this study, the differential cerebellar regions identified are the Left lobule VI (GM) and Right Crus II (WM), diverging from findings of prior reports.”

The corrected sentence appears below:

“In this study, the differential cerebellar regions identified are the Left lobule VI (GM), diverging from findings of prior reports.”

In the published article, there was an error. A correction has been made to **Discussion**, *Fifth paragraph*. This sentence previously stated:

“In our study, we found that the Right Crus II was the area that exhibited volume differences in white matter between the two groups. However, this difference was not significantly associated with cognitive evaluation scores, including MMSE and MoCA. TLE patients may experience abnormal neuronal discharges (Engel, 1983), which can potentially damage the neural cells and fibers in the cerebellum. This damage may result in a reduction in cerebellar white matter volume. The Crus II of the cerebellum is involved in language processing (Gelinas et al., 2014; Riva et al., 2019), specifically in the semantic processing of sentences (D'Mello et al., 2017; Nakatani et al., 2022).”

The corrected sentence appears below:

“In this study, after FDR correction, we did not identify any significant differences in the cerebellar white matter between the two groups. It may be necessary to continue expanding the sample size for further research.”

The authors apologize for these errors and state that they do not change the scientific conclusions of the article in any way. The original article has been updated.

